# siRNA against CD40 delivered via a fungal recognition receptor ameliorates murine acute graft‐versus‐host disease

**DOI:** 10.1002/jha2.439

**Published:** 2022-05-06

**Authors:** Beate Heissig, Yousef Salama, Masatoshi Tateno, Satoshi Takahashi, Koichi Hattori

**Affiliations:** ^1^ Department of Research Support Utilizing Bioresource Bank Graduate School of Medicine Juntendo University School of Medicine Tokyo Japan; ^2^ An‐Najah Center for Cancer and Stem Cell Research Faculty of Medicine and Health Sciences An‐Najah National University Nablus Palestine; ^3^ Department of Pathology Kushiro Red Cross Hospital Kushiro Hokkaido Japan; ^4^ Division of Clinical Precision Research Platform Institute of Medical Science University of Tokyo Tokyo Japan; ^5^ Center for Genomic & Regenerative Medicine Juntendo University School of Medicine Tokyo Japan

**Keywords:** acute GvHD, bone marrow transplantation, CCR2, CD40, cytokine, Dectin1, IL17A, PAMP, siRNA

## Abstract

Acute graft‐versus‐host disease (aGvHD) remains a major threat to a successful outcome after allogeneic hematopoietic stem cell transplantation (HSCT). Although antibody‐based targeting of the CD40/CD40 ligand costimulatory pathway can prevent aGvHD, side effects hampered their clinical application, prompting a need for other ways to interfere with this important dendritic T‐cell costimulatory pathway. Here, we used small interfering RNA (siRNA) complexed with β‐glucan allowing the binding and uptake of the siRNA/β‐glucan complex (siCD40/schizophyllan [SPG]; chemical modifications called NJA‐312, NJA‐302, and NJA‐515) into Dectin1+ cells, which recognize this pathogen‐associated molecular pattern receptor. aGvHD was induced by the transplantation of splenocytes and bone marrow cells from C57BL/6J into CBF1 mice. Splenic dendritic cells retained Dectin1 expression after HSCT but showed lower expression after irradiation. The administration of siCD40/SPG, NJA‐312, and NJA‐302 ameliorated aGvHD‐mediated lethality and tissue damage of spleen and liver, but not skin. Multiple NJA‐312high injections prevented aGvHD but resulted in early weight loss in allogeneic HSCT mice. In addition, NJA‐312 treatment caused delayed initial donor T and B‐cell recovery but resulted in stable chimerism in surviving mice. Mechanistically, NJA‐312 reduced organ damage by suppressing CCR2+, F4/80+, and IL17A‐expressing cell accumulation in spleen, liver, and thymus but not the skin of mice with aGvHD. Our work demonstrates that siRNA targeting of CD40 delivered via the PAMP‐recognizing lectin Dectin1 changes the immunological niche, suppresses organ‐specific murine aGvHD, and induces immune tolerance after organ transplantation. Our work charts future directions for therapeutic interventions to modulate tissue‐specific immune reactions using Pathogen‐associated molecular pattern (PAMP) molecules like 1,3‐β‐glucan for cell delivery of siRNA.

## 1 INTRODUCTION

Allogeneic hematopoietic stem cell transplantation (HSCT) is a curative therapy for many immune and hematologic malignant and non‐malignant disorders. Once transplanted, allogeneic cells support myeloid and immunological reconstitution, and require a degree of immune tolerance against allogeneic antigens. Allogeneic T cells contained in the transplant attack and destroy the recipient organs including intestine, liver, and skin contributing to graft failure, and graft‐versus‐host disease (GvHD).

Acute GvHD (aGvHD) is a major cause of morbidity and mortality in allogeneic HSCT patients [1]. The conditioning regimen‐mediated tissue damage accelerates the release of inflammatory cytokines. Cytokines activate recipient and donor‐derived antigen presenting cells (APCs) that expand alloreactive donor T cells with the help of costimulatory signals like CD40/CD40 ligand (CD40L, CD154). CD40 engagement on the surface of APCs like dendritic cells (DCs) by CD40L on activated CD4+ helper T cells further enhances cytokine production and induces costimulatory molecules on their surface that facilitates the cross‐presentation of antigen [2, 3, 4]. CD40 is expressed on B cells, DCs, monocytes/macrophages, platelets, and epithelial or endothelial cells [5, 6].

Targeting the costimulatory receptor/ligand CD40/CD40L signaling prevents antigen‐specific T‐cell receptor activation, reduces aGvHD severity, and induces organ tolerance in preclinical and clinical studies [7, 8, 9]. Although antibody‐mediated inhibition of CD40L induced CD4 T‐cell tolerance to host alloantigens and reduced GvHD [10, 11], clinical studies were halted due to thromboembolic complications after antibody administration [12, 13, 14]. Given the importance of CD40 as an anti‐GvHD target, we searched for other ways to block the CD40/CD40L costimulatory pathway.

Interrupting the CD40/CD40L signal can be achieved by silencing CD40 gene expression using small interfering RNA (siRNA). These short double‐stranded RNA sequences quench target gene expression. To overcome the challenge of rapid siRNA degradation in circulation before reaching target cells siRNA compounds were prepared whereby siRNA via its poly‐dA extension at the 5′‐end of the siRNA binds fungal (schizophyllan [SPG]) 1,3‐β‐glucan [15, 16, 17]. The siRNA‐β‐glucan complex like the native 1,3‐β‐glucan or galectins, and annexins on apoptotic cells can interact with the cellular‐anchored DC‐associated C‐type lectin (Dectin1, CLEC‐7A). Monocytes, or conventional and plasmacytoid DCs mainly express Dectin1 [18]. The Dectin1‐β‐glucan/siRNA complex interaction takes advantage of the fact that APCs express Dectin1 and that the complex via Dectin1 is internalized into lysosomal endosomes ensuring that attached cargo is incorporated into the cell. Here, we tested a siRNA targeting CD40 complexed with SPG (siCD40–SPG) that targets CD40 expression on Dectin1+ cells in a lethal murine allogeneic bone marrow transplantation (BMT) model.

## 2 METHODS

### 2.1 Reagents

siRNAs target mouse CD40 and human CD40, respectively [19]. All siRNAs contained polydeoxynucleotide consisting of 40 adenosines (dA40) at the 5′ end of the sense strand. siRNA with dA40 for CD40 (sequence: 5′‐UGAUUCUGCGGUGCCCUCC‐3′) denoted as NJA‐312 or NJA‐302 or NJA‐515 (sequence: 5′‐UACUGUUUGUCACUGCACG‐3′) were synthesized by Gene Design Co., Ltd. (Osaka, Japan) and purified by high‐performance liquid chromatography. The compounds were kindly provided by NapaJen Pharma Co., Inc. (Tokyo, Japan).

#### 2.1.1 Mice

Female C57BL/6 (B6; H‐2b) and (BALB/c × C57BL/6) F1 (CBF1; H‐2b/d) recipient mice were purchased from SLC (Shizuoka, Japan) and used for experiments at the age of 10 weeks (bodyweight 20 g) [20, 21].

#### 2.1.2 Induction of lethal aGvHD

Before transplantation, CBF1 mice were irradiated with 8.0 Gy using a Co irradiator (MBR 1505 R, Hitachi, Tokyo, Japan), fractionated into 4.0 Gy doses at 4 h intervals. The irradiation group did not receive further BMT. BMT model: GvHD was induced in irradiated CBF1 mice by intravenous (i.v.) injections of 1 × 10^7^ bone marrow (BM) cells and 5 × 10^7^ spleen cells from B6 mice on day 0 (allogeneic). Irradiated CBF1 mice were injected with 1 × 10^7^ BM cells and 5 × 10^7^ spleen cells from CBF1 mice (syngeneic). Day 0 defined the day of BMT.

#### 2.1.3 Treatment group

**FIGURE 1:**
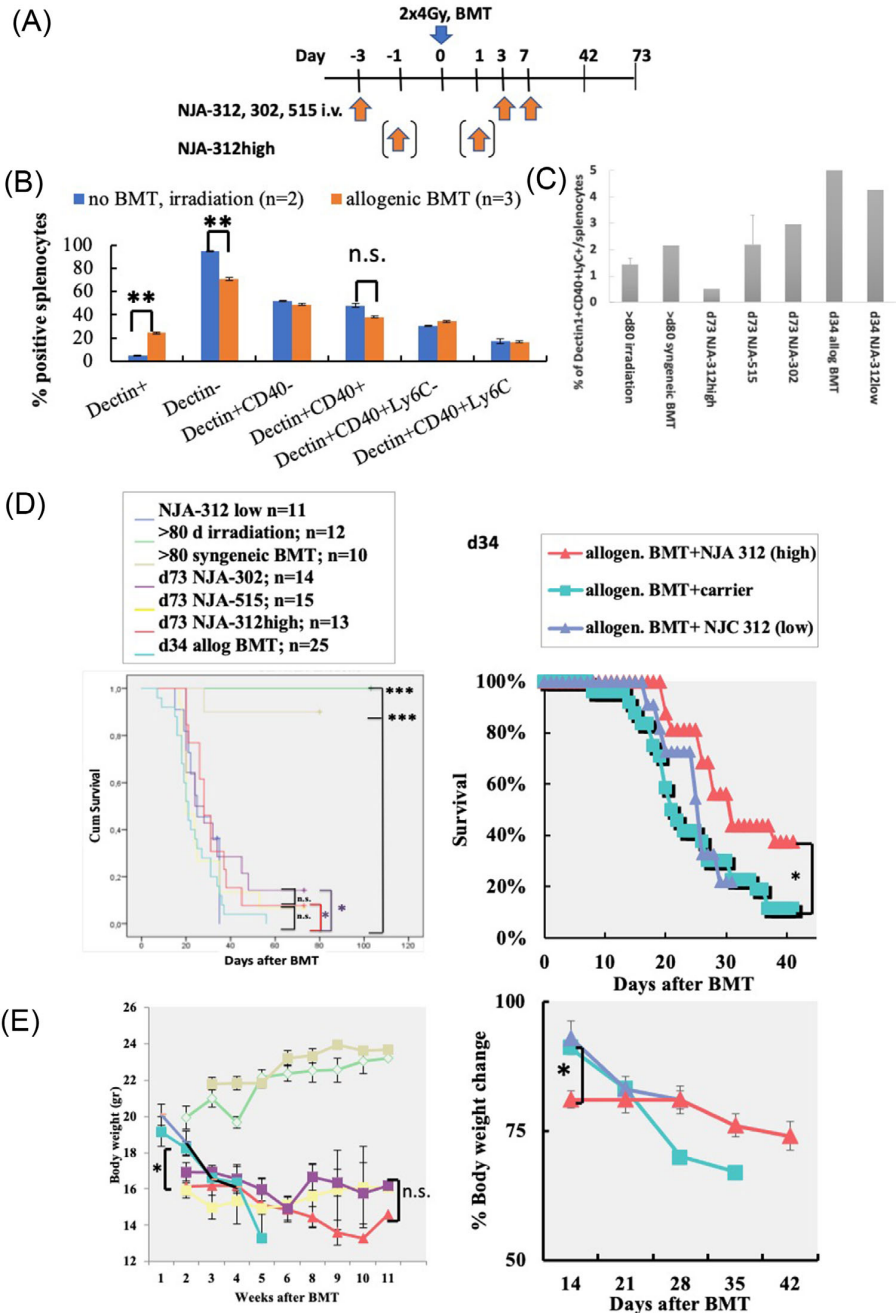
Reduced graft‐versus‐host disease (GvHD) lethality in mice treated with small interfering RNA (siRNA) against CD40. (A) Experimental protocol of allogenic bone marrow transplantation (BMT): all mice received 8 Gy total body irradiation. In the allogeneic BMT group, F1 mice were given B6 donor splenocytes and BM cells. F1 mice received F1 splenocytes and BM cells to establish the syngeneic BMT group. Allogenic BMT mice were treated either with/without siRNA targeting mouse CD40 (siCD40/schizophyllan [SPG]) called NJA‐312, NJA‐302, or NJA‐515. (B) Splenocytes retrieved at day 34 after BMT were co‐stained with antibodies against Dectin1, CD40, and Ly6c. Percentages of Dectin+ and Dectin+CD11c‐ cells were determined by three‐color flourescence‐activated cell sorting (FACS) in splenocytes from mice receiving 8 Gy (irradiation, *n* = 2) without BMT (noBMT, 8 Gy, *n* = 3). (C) Splenocytes retrieved at indicated time points and treatment groups (after NJA‐312, NJA‐302, and NJA‐515 treatment) were analyzed by FACS using the following antibodies: anti‐Dectin1, anti‐Ly6c, and anti‐CD40 (*n* = 1–2/group). (D) Survival of mice undergoing indicated treatments was monitored daily. Data were plotted as a Kaplan–Meier curve. Statistics using SPSS software using log‐rank (Mantel‐Cox). Left panel: indicated groups are shown (same data as left panel). The number of mice per group were as follows: *NJA‐312low (*n* = 11); >80 d irradiation (*n* = 12); >80 syngeneic BMT (*n* = 10); d73 NJA‐302 (*n* = 14); d73 NJA‐515 (*n* = 15); d73 NJA‐312high (*n* = 13); d34 allog BMT (*n* = 25). (E) Bodyweight was determined weekly (*n* = 7 per treatment group). Left panel: all groups are shown. Right panel: only indicated groups are shown (same data as left panel). *p < 0.05; **p < 0.01, ***p < 0.0001

Controls were injected with Phosphate‐Buffered Saline (PBS)/carrier. siRNA injections like NJA‐515, NJA‐302, and NJA‐312 were injected i.v. on days ‐3, +3, and +7 (low dose), or on days ‐3, ‐1, +1, +3, and +7 (high dose) as for NJA‐312 (2 μg/200 μl PBS/mouse/time point; Figure 1A). NJA‐312low (*n* = 11); irradiation (*n* = 12); >80 syngeneic BMT (*n* = 10); NJA‐302 (*n* = 14); NJA‐515 (*n* = 15); NJA‐312high (*n* = 13).

#### 2.1.4 Flow cytometric analysis

Cells (1 × 10^6^) were stained with Fluorescein isothiocyanate Fluorescein isothiocyanate (FITC)‐conjugated anti‐mouse Ly6c (AbD Serotec, Duesseldorf, Germany), FITC‐conjugated H2kd, Phycoerythrin (PE)‐conjugated anti‐H2kb, PE‐conjugated anti‐CD40, PE‐conjugated anti‐CD4, FITC‐conjugated anti‐CD8a, APC‐conjugated anti‐B‐220, APC‐conjugated anti‐CD369 (PharMingen, San Diego, CA, USA), and APC‐conjugated CD3e and analyzed on a FACSCalibur.

#### 2.1.5 GvHD histopathology

Tissues were harvested from—two to three mice per group on days 34 and 73 after BMT, fixed in 4%‐buffered freshly prepared paraformaldehyde, and embedded in paraffin. Hematoxylin and eosin (H&E) staining of the paraffin‐embedded tissue sections was performed according to standard protocols. The stained sections were evaluated in a blinded fashion by a pathologist and images were taken using an OLYMPUS microscope (Eclipse, TE 300; Melville, NY, USA).

### 2.2 Immunohistochemistry

Automated immunohistochemistry was performed using the VENTANA BenchMark GX (Ventana Medical Systems Inc., Tucson, AZ, USA). Deparaffinization, peroxidase inhibition, and antigen retrieval was performed (CC1: 1 mM Ethylenediaminetetraacide acid (EDTA), pH 8.5). According to the manufacturer's protocol, the slides were stained with the primary Ab overnight at 4°C and counterstained. Primary Abs included rat anti‐mouse CCR2 Ab (clone FAB538A, R&D Systems), rabbit anti‐mouse CD11b (GTX134542, GenTex), rabbit anti‐mouse/human F4/80 (PA5‐32399), goat anti‐rabbit IL17A (Abcam, Cambridge, UK, cat no. ab79056), rabbit anti‐mouse CD8e (cat no. 550281, BD Pharmingen), and rabbit anti‐mouse/human/rat CD3 (clone 5690, abcam). Secondary antibodies included rabbit anti‐rat IgG (for CCR2 and F4/80) and goat anti‐rabbit (for CD11b, CD8a, CD3). Slides were developed using the iView DAB universal Kit (code 100032, Ventana Medical Systems Inc.) or ultraView Universal Alkaline Phosphatase Red Detection Kit (#760‐501, Ventana Medical Systems Inc.).

#### 2.2.1 Data sharing statement

For original data, please contact heissig@juntendo.ac.jp.

#### 2.2.2 Statistical analysis

We plotted the survival curves using Kaplan—Meier estimates. We used the log‐rank test for the analysis of survival data. The Student's *t*‐test was used for the statistical analysis of other data. Data are presented as means ± S.E.M. *p* < 0.05 was considered statistically significant.

## 3 RESULTS

### 3.1 Dectin1+ cells coexpress CD40 in aGvHD tissues

Because CD40 expression by DCs enhances aGvHD, we reasoned that reducing the expression of CD40 on Dectin1+ DCs [19] siCD40–SPG compounds would suppress aGvHD in murine allogeneic BMT models. We established a murine major histocompatibility complex‐mismatched BMT model of lethal aGvHD by injecting BM cells and splenocytes from C57/Bl6 mice i.v. into irradiated (2 × 4 Gy) CBF1 mice (Figure 1A). The BM/splenocyte transplantation was performed on day 0. Groups of mice receiving irradiation without further cell transplantation (irradiation group) or irradiation followed by the transplantation of CBF1 mouse‐derived BM cells and splenocytes (syngeneic group) were included to control for irradiation or non‐GvHD, but transplantation‐procedure‐associated effects. Because the siRNA compounds require the presence of Dectin1 to enter cells, Dectin1 expression was examined on splenocytes of mice >80 days after irradiation or mice 34 days after allogeneic BMT (Figure 1B). After irradiation, Dectin1 expression on splenocytes was lower compared to mice 34 days after allogeneic BMT as determined by FACS (Figure 1B). Further characterization showed that roughly 50% of Dectin1+ cells co‐stained for CD40 (Figure 1B). Within the Dectin‐1+ cell population that coexpressed CD40, around 10% of cells also co‐stained with Ly6C, an antigen that can be found on macrophage/DC precursors (Figure 1B). These data confirmed that 50% of Dectin1+ splenocytes coexpressed CD40 after allogeneic BMT and that these cells contained a subpopulation of Ly6C+ macrophage/DC precursors.

Two of the tested siRNA compounds targeted the same gene sequence but differed in their chemical modifications (NJA‐312 and NJA‐302), while one targeted a different CD40 sequence (NJA‐515). Two administration schedules were tested for the compound NJA‐312: three injections (referred to as NJA‐312low) and five injections (NJA‐312high).

The frequency of Dectin1+CD40+Ly6C+ splenocytes was similar between NJA‐312low and carrier‐treated mice after allogenic BMT controls on day 34 (Figure 1C). In surviving mice by day 73, a lower frequency of Dectin1+CD40+Ly6C+ cells was observed in NJA‐312high‐treated splenocytes of allogeneic BMT mice compared to syngeneic BMT controls (Figure 1C). These data suggested that administration of NJA‐312low did not change the frequency of Dectin1+CD40+Lyc+ splenocytes and that NJA‐312high suppressed Dectin1+CD40+Lyc+ splenocytes in long‐term surviving mice.

### 3.2 NJA‐312 prolong survival in allogenic BMT mice

Survival and bodyweight loss are reliable indicators of aGvHD in murine models [20]. CBF1 mice transplanted with CBF1 cell following irradiation served as controls (syngeneic BMT). In the syngeneic group, all mice survived. Few or no mice survived long‐term in this lethal aGvHD model where mice had been transplanted with allogenic hematopoietic cells of C57BL6 cell origin (allogeneic BMT). NJA‐302 and NJA‐312, but not NJA‐515 showed similar survival (data not shown). The analysis was continued using NJA‐312. NJA‐312high, but not NJA‐312low injections prolonged survival compared to carrier‐injected allogeneic BMT mice (Figure 1D). Long‐term surviving mice still showed a lower bodyweight when compared to syngeneic BMT or irradiation controls (Figure 1E). NJA‐312high, but not NJA‐312low‐injected mice showed bodyweight loss during the first 14 days (Figure 1E), suggesting that dose escalation enhanced survival at the cost of bodyweight loss.

### 3.3 Improved thymic regeneration after NJA‐312high treatment in aGvHD mice

**FIGURE 2:**
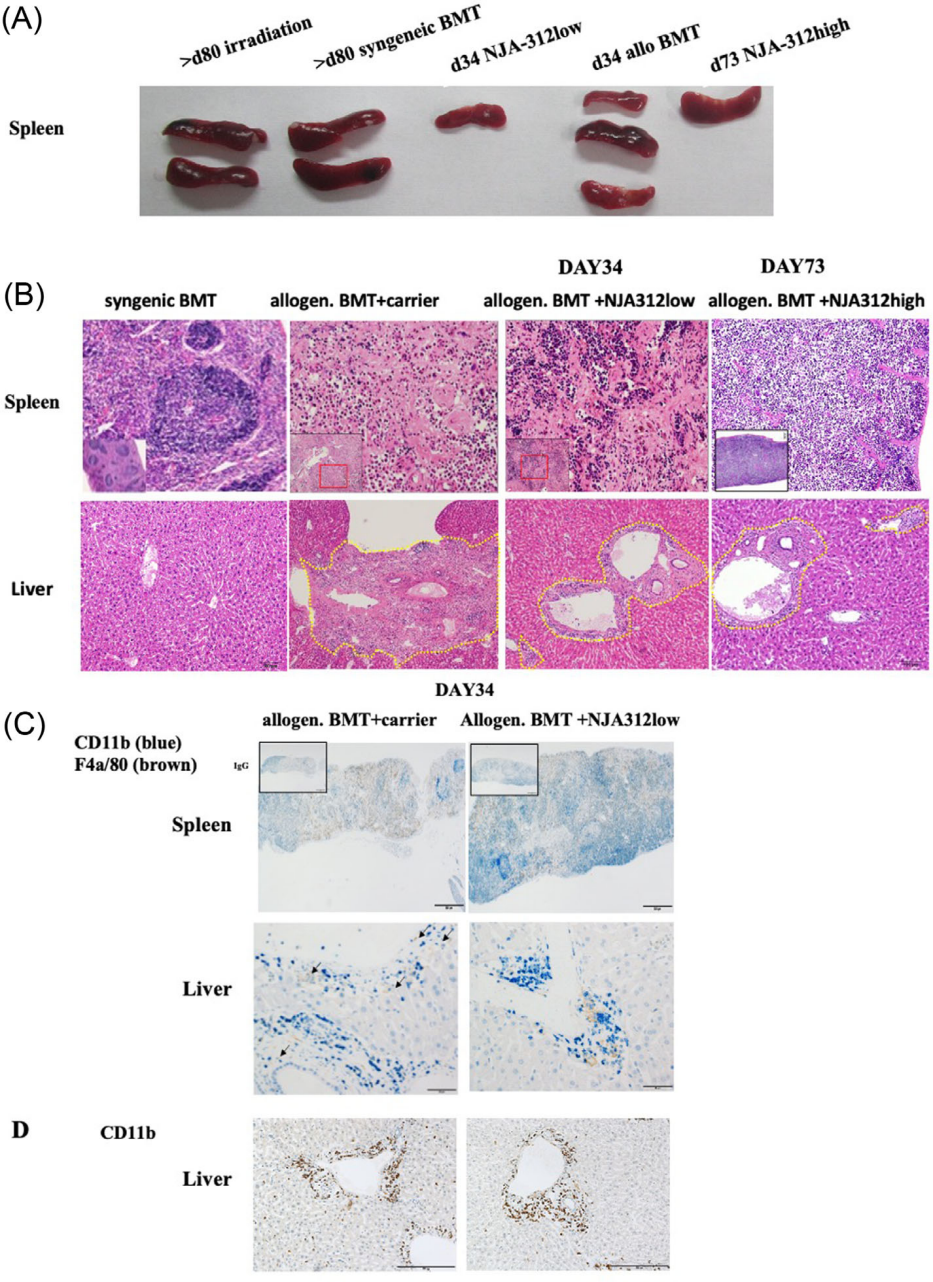
NJA‐312 treatment does not prevent acute thymus atrophy but results in normal T‐cell development in surviving mice. (A) Hematoxylin and eosin (H&E)‐stained thymus sections of indicated treatment groups and time points (magnification bar = 200 μm). (B) Percentage of cells expressing CD4‐CD8+, CD4+CD8+, and CD4+CD8‐ on thymocytes as analyzed by FACS. Only live thymocytes, as defined by propidium iodide exclusion were analyzed (*n* = 2–3 per group). (C) Immunoreactive CD11b+ c (blue signal) and F4/80+ cells (brown signal) on thymus sections 34 days after allogeneic bone marrow transplantation (BMT) in the carrier‐ and NJA‐312low‐treated mice (magnification bar = 50 μm). Inserts in (D) show isotype controls (bar = 100 μm)

Carrier and NJA‐312‐treated allogeneic BMT mice showed typical signs of aGvHD in the thymus with atrophy and hypocellularity by day 34 (Figure 2A). Surviving NJA‐312high‐treated mice showed a nearly complete restoration of the thymic architecture by day 72, indicative of thymic regeneration.

Earlier studies showed that blockade of CD40/CD40L interaction prevents GvHD by reducing donor CD4+ but not CD8+ T‐cell expansion [22]. We assessed the intrathymic T‐cell development. On day 34 after BMT, the relative distribution of thymocytes subpopulations was abnormal (Figure 2B). The proportion of double‐positive (DP, CD8+CD4+) cells and single‐positive (SP) CD4 cells was greatly diminished in both carrier and NJA‐312low‐treated allogeneic BMT mice. Viable thymocytes consisted nearly exclusively of SP CD8+ cells. By day 73, similar to the syngeneic BMT group, the thymocytes showed a distribution pattern with mostly DP CD8+CD4+ cells, while SP CD4+ and CD8+ cells made up around 28% and 10%, respectively, of the whole thymocyte population (Figure 2B), indicating that thymic regeneration had occurred in the surviving NJA‐312high mice.

### 3.4 Delayed T and B‐cell engraftment, but stable lymphoid chimerism of long‐term survivors after NJA‐312 treatment

Given that aGvHD results in a severe anti‐allogeneic CTL response, we next used FACS analysis to examine the percentage of CD3+ and B220+ lymphocytes of recipient (H‐2K^d+^K^b+^) or donor (H‐2K^d−^K^b+^) origin in splenocytes of allogenic BMT mice treated with or without NJA‐312low (Table 1). By day 34, we observed a trend toward a delayed CD3 T and B220 B donor cell engraftment in vivo in NJA‐312‐treated, compared to carrier‐treated, allogenic BMT mice. This prompted us to also analyze T and B cells in the surviving mice on day 73 (Table 1). Therefore, on day 73, with no carrier‐treated mice available for analysis, we compared T‐cell reconstitution between NJA‐312high‐treated allogeneic BMT mice and irradiated mice that did not receive BMT (Table 1). In the surviving NJA‐312high‐treated recipients, 31.8% of B220+ and 62% of CD3+ splenocytes were H‐2K^b+^ and H‐2K^d−^ and therefore of donor origin, indicating partial replacement of the recipient lymphoid system by donor cells and establishment of donor–recipient chimerism (Table 1).

**TABLE 1:** Identification of donor‐derived cells within the recipient lymphoid organs

	d34			d73		
Splenocytes	Allog BMT + carrier (*n* = 3)	Allog BMT + CD40 siRNA (*n* = 1)	*p* ‐Value	Irradiation or syngeneic BMT controls (*n* = 2–3)	Allog BMT + CD40 siRNA (*n* = 3)	*p* ‐Value
% Donor B220^+^ H‐2K^d‐^K^b+^	44.6 (+/‐20.7)	13.8	n.a.	0.63 (+/‐0.2)	31.8 (+/‐14.7)	<0.05
% Recipient B220^+^ H‐2K^d+^K^b+^	54.8 (+/‐20.8)	82.4	n.a.	98.1 (+/‐0.8)	52.4 (+/‐14.7)	<0.05
% Donor CD3^+^ H‐2K^d‐^K^b+^	47.5 (+/‐20.2)	23.9	n.a.	0.3 (+/‐0.05)	62 (+/‐8.9)	<0.001
% Recipient CD3^+^ H‐2K^d+^K^b+^	52.0 (+/‐20.0)	74.2	n.a.	99.4 (+/‐0.05)	33.2 (+/‐13)	<0.001

*Note*: FACS analysis of CD3e and B220+ stained splenocytes co‐stained with H‐2K^b^ and H‐2K^d^ at indicated days after BMT. Donor‐derived cells (H‐2K^d‐^K^b+^) were identified within the recipient spleen cells (H‐2K^d+^K^b+^) by three‐color flow cytometry.

Abbreviations: BMT, bone marrow transplantation; siRNA, small interfering RNA.

### 3.5 NJA‐312‐treated mice show less tissue damage in the spleen and liver

**FIGURE 3:**
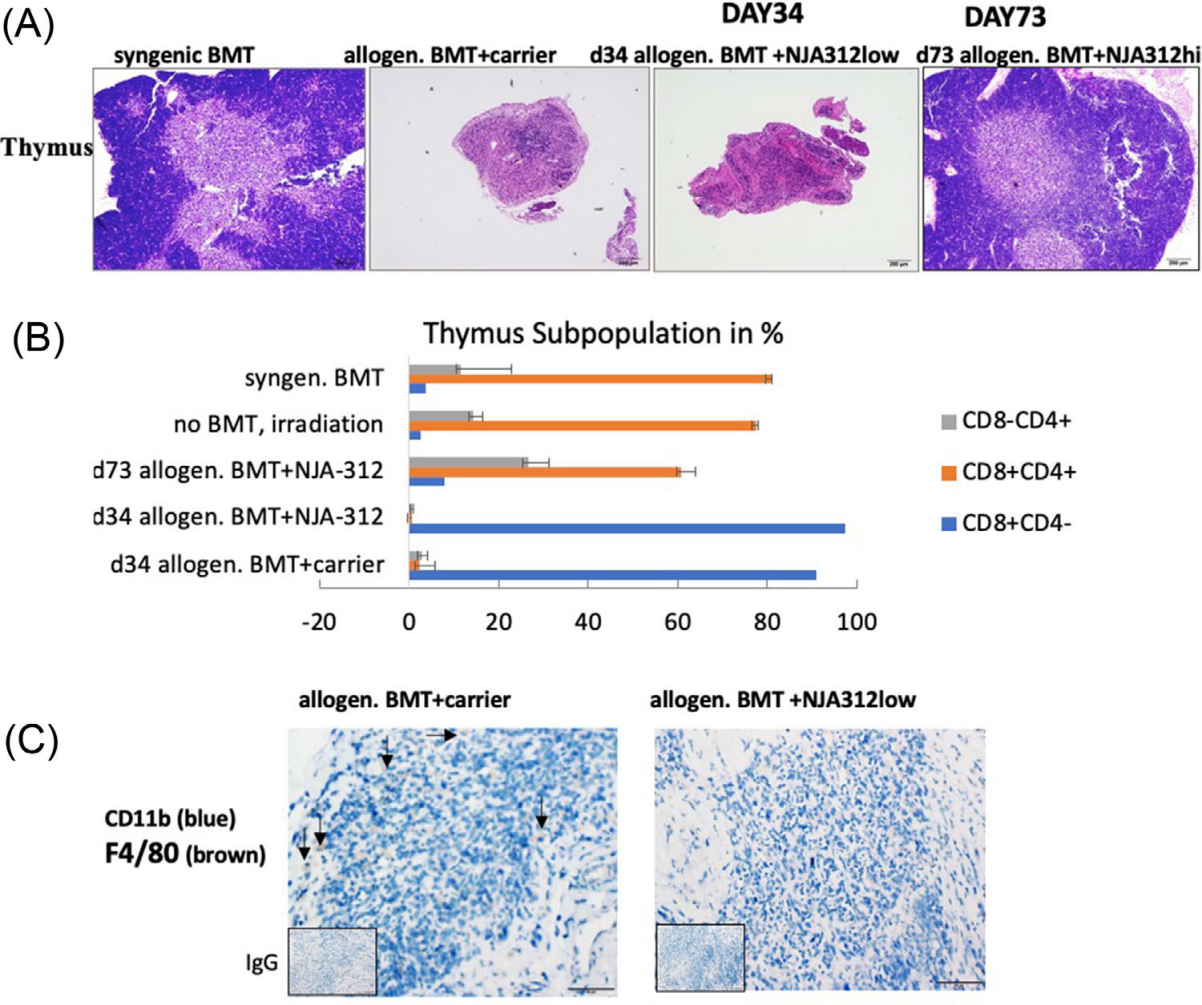
Treatment with NJA‐312 impairs liver and spleen organ damage in graft‐versus‐host disease (GvHD) mice. (A) Macroscopic images of spleens of different treatment groups at indicated time points. (B) Hematoxylin and eosin (H&E)‐stained spleen and liver tissue sections of different treatment groups at indicated time points. Magnification of main image (bar = 100 μm), inserts (bar = 100 μm). Yellow dotted lines encircle periportal areas with fibrosis and leukocyte infiltrates. (C) On day 34 immunoreactive CD11b (blue signal) and F4/80 (brown signal) were determined on liver sections of allogenic bone marrow transplantation (BMT) mice treated with carrier or NJA‐312 and representative images are shown (bar = 100 μm). Insert image shows isotype staining (bar = 200 μm). (D) Representative images of immunoreactive CD11b cells in liver tissue sections of allogenic BMT mice treated with carrier or NJA‐312 (magnification bar = 200 μm)

Dectin‐1 mRNA is predominantly detected in the spleen and thymus DCs [23]. Smaller spleens were found in the carrier‐ and NJA‐312low‐treated allogeneic BMT compared to post‐irradiation or syngeneic BMT mice (Figure 3A) indicating aGvHD‐driven tissue damage even after NJA‐312low treatment. Histopathological findings revealed marked lymphoid atrophy, hypocellularity, fibrosis, and disappearance of lymphoid follicles in spleens of carrier‐ and NJA‐312low‐treated allogeneic BMT mice. NJA‐312low‐derived spleens retained higher splenic cellularity (Figure 3B). Long‐term surviving mice showed an average spleen size and no pathological histological findings (Figure 3A,B). In the H&E‐stained liver sections of control mice, massive infiltration of mononuclear cells and total scar were observed. In the liver of NJA‐312low‐treated mice, periportal infiltration of mononuclear cells and fibrosis was found (Figure 3B).

To examine the extent of myeloid cell infiltration, especially monocytes, macrophages, granulocytes, and natural killer cells, immunohistochemistry with antibodies against CD11b (blue signal) and F4/80 (brown staining) (Figure 3C) and only CD11b alone (Figure 3D) were performed. The number of CD11b+F4/80+ and periportal CD11b+ cells was similar in liver tissues of both groups (Figure 3C,D). These data implicated that NJA‐312low treatment suppressed myeloid F4/80+ cell expansion in the spleen and liver.

Because severe aGvHD and a Th1‐skewed inflammatory marrow environment suppress hematopoiesis and donor HSC engraftment [24], we examined BM sections. Local hypocellularity was observed in one out of three BM sections of the carrier‐treated, but not NJA‐312low‐treated aGvHD mice at day 34 after BMT (Figure S1B) indicating that NJA‐312 treatment was not myelosuppressive and did not cause graft failure.

### 3.6 Skin GvHD and CD11b+, CCR2+ dermal infiltrates are unaltered by NJA‐312 treatment

**FIGURE 4:**
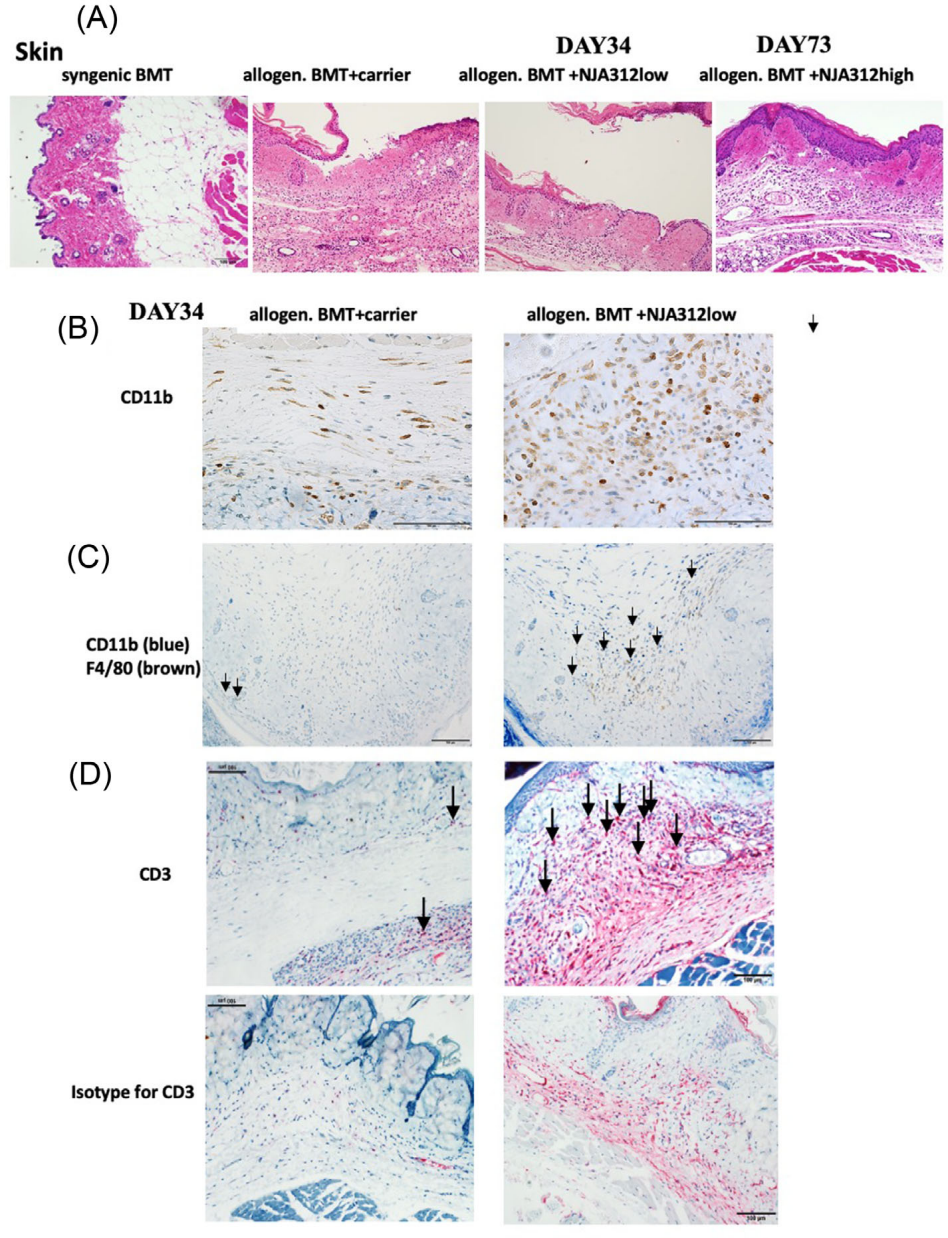
Targeting CD40 using the small interfering RNA (siRNA) compounds did not prevent skin acute graft‐versus‐host disease (aGvHD). (A) Hematoxylin and eosin (H&E)‐stained liver and spleen tissue sections of different treatment groups at indicated time points. Magnification of main image (bar = 100 μm). (B) Representative images of immunoreactive CD11b cells in liver tissue sections of allogenic bone marrow transplantation (BMT) mice treated with carrier or NJA‐312 (magnification bar = 100 μm). (C) Representative images of skin sections from animals 34 days after BMT stained with CD11b (blue signal) and co‐stained with F4/80 (brown signal) (bar = 100 μm). Arrows indicate examples of positive cells in both sections. (D) Representative images of skin sections from animals 34 days after BMT stained with CD3 (red signal) in the upper panels (bar = 100 μm). Arrows indicate examples of CD3+ cells in all images. Isotype controls are given

Histopathological examination revealed blister formation and inflammation in one of the sections derived from carrier‐treated allogenic BMT mice. NJA‐312‐treated allogeneic BMT mice showed crust formation and acantholysis with deep dermal inflammation by day 34 (Figure 4A). No pathological changes were observed in surviving mice treated with NJA‐312high at day 72 (Figure 4A).

Immunohistochemical analysis revealed increases in immunoreactive CD11b+, F4/80+, and CD3+ cells in the dermis of NJA‐312low‐treated, compared to carrier‐treated, allogenic BMT mice (Figure 4B–D). These data indicate that NJA‐312 was less effective in suppressing the generation of inflammatory infiltrates in the skin, perhaps due to the low Dectin1 expression reported in skin macrophages and DCs [25]. It has been reported that Dectin1 on macrophages promotes intestinal inflammation [26]. No pathological changes were observed in colon tissues of carrier and NJA‐312 mice (Figure S1A).

### 3.7 siCD40 compounds enhanced expansion of CD8+ cells in the spleen and thymus, but not the skin

**FIGURE 5:**
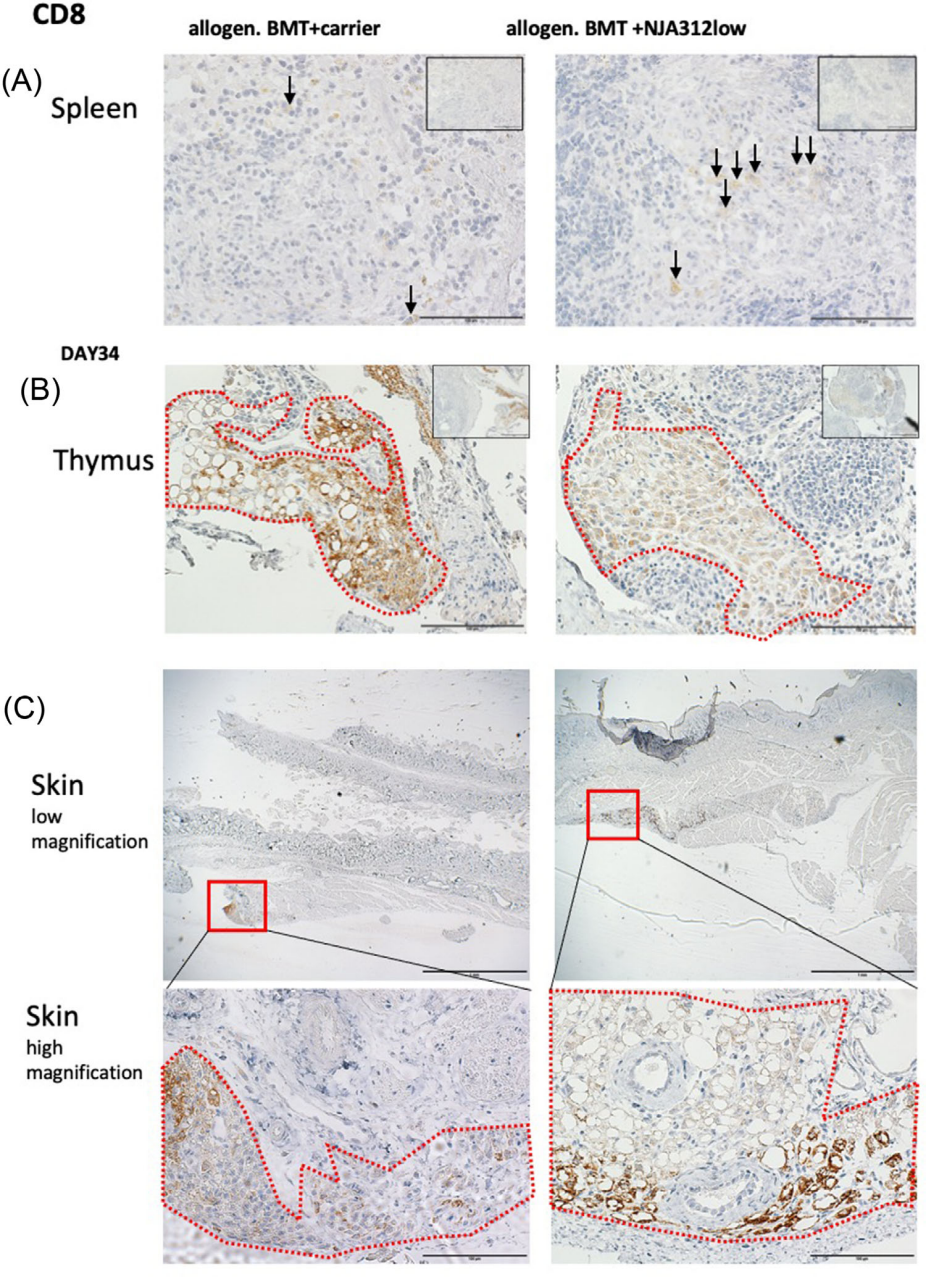
Focal expansion of large CD8+‐expressing cells in thymi of NJA‐312low‐treated mice, but not the skin. (A–C) Representative images of immunoreactive CD8a in the spleen (A), thymus (B), and skin (C) in tissues were retrieved on day 34 after bone marrow transplantation (BMT). Red dotted lines encircle areas of CD8+ cell expansion (upper panels magnification bar = 1 mm; lower panels magnification bar = 100 μm). Arrows indicate examples of positive cells in sections

Both host and donor DCs contribute to inducing donor T‐cell tolerance against host tissues in mice undergoing allo‐HSCT. CD8+ DCs have been described in both the thymus and spleen, constituting 70% of the thymus DCs and 20% of the spleen DCs that can express Dectin1. These cells suppress donor T‐cell response and impair severe GvHD [27]. We performed CD8a staining on spleen and thymus tissue sections 34 days after allogeneic BMT. CD8+ cells were increased in spleen and thymus tissues of NJA‐312‐treated mice. The CD8+ cells, especially in the thymus and skin, presented as large cells with round nuclei, centrally located nucleoli, bland and dispersed chromatin, and flattening of adjacent nuclear membrane, suggesting that they might be DCs. The dermis of carrier‐ and NJA‐312‐treated animals showed similar numbers of infiltrating CD8+ cells (Figure 5C). These data indicate that CD8+ cell expansion occurred primarily in the thymus, but not the skin, of NJA‐312‐treated mice.

### 3.8 Reduced number of CCR2+ cell infiltrates in spleen and liver of NJA‐312‐treated mice

**FIGURE 6:**
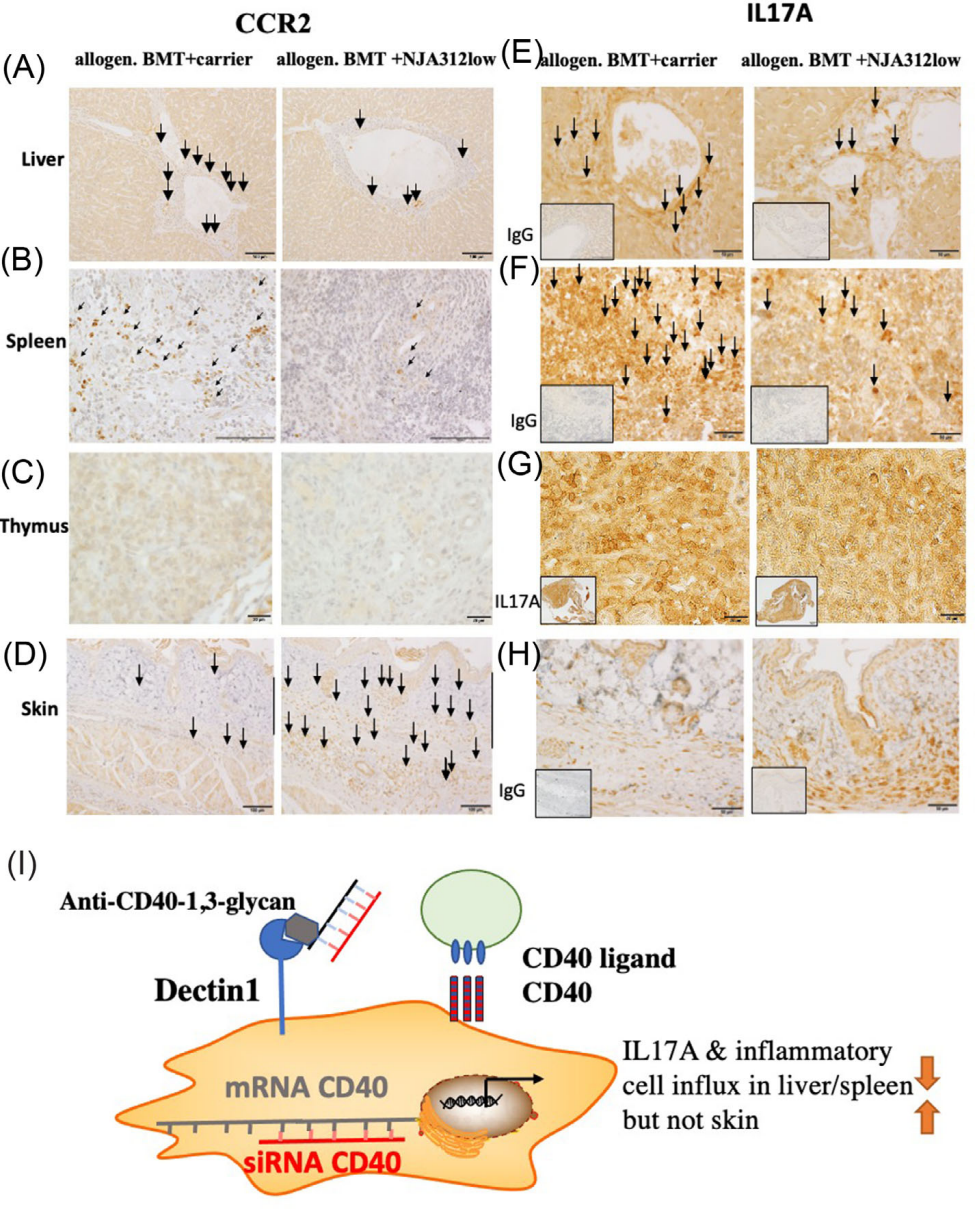
NJA‐312low treatment altered IL17A and CCR2 expression in an organ‐specific manner in acute graft‐versus‐host disease (aGvHD) tissues. (A–D) Representative images of CCR2‐stained tissues section of the liver (A), spleen (B), thymus (C), and skin (D) of the carrier‐ or NJA‐312‐treated allogeneic bone marrow transplantation (BMT) mice. Immunoreactivity for CCR2 (E–G) in sections of liver (E), spleen (F), thymus (G), and skin (H) is depicted as brown staining. Strongly IL17A‐expressing cells were indicated using arrows. Magnification of spleen and liver (bar = 50 μm) and skin (bar = 20 μm) images. Inserts show isotype controls in (E), (F), and (H) (bar = 1 mm). The (G) insert gives a low magnification of IL17A‐stained thymus sections. (I) siCD40/schizophyllan (SPG) can incorporate into Dectin1+ cells where it reduces CD40 expression on CD40+ cells like myeloid cells (macrophages or dendritic cells [DCs]) thereby preventing further costimulation of T cells via CD40/CD40L interaction

The interactions between CCR2 and CCL2 are involved in T‐cell/monocyte/macrophage migration resulting in the accumulation of CCR2+ cells in aGvHD tissues [21]. In addition, Dectin1 is crucial for Ly6ChighCCR2high monocyte population enrichment in the blood and their recruitment to the inflamed colon as precursors of inflammatory macrophages [26]. Tissue sections were stained using CCR2 antibodies to investigate the influence of NJA‐312 treatment on the cellular composition of tissue‐residing mononuclear cells. Thymic cells derived from NJA‐312‐treated mice had fewer CCR2+ cells in periportal areas of the liver and throughout the spleen (Figure 6A,B), and a reduced number of cells strongly stained for CCR2 (Figure 6C). In contrast, skin sections of NJA‐312‐treated mice showed more infiltrating CCR2+ cells in the dermis (Figure 6D). These data indicated that NJA‐312 treatment reduced the CCR2+ cell influx into the liver and spleen, but not skin.

### 3.9 IL17A expression is modulated in hematopoietic and non‐hematopoietic cells in aGvHD tissues

Dectin1 stimulation during Candida infection induces Th17 responses [28]. Studies on the role of IL17A are controversial with some indicating IL17A as a promoter or suppressor of GvHD [29, 30]. Therefore, we assessed IL17A expression in aGvHD‐prone tissues. While IL17A expression under non‐pathological conditions is found mainly on T cells (data not shown), hematopoietic and non‐hematopoietic cells in tissue sections after BMT expressed IL17A (Figure 6E–H). NJA‐312 treatment reduced the intensity of IL17A expression in the liver, spleen, and thymus (Figure 6E–G) with hematopoietic cells showing strong, and non‐hematopoietic‐like hepatocytes showing weak, IL17A expression by immunohistochemistry staining. In contrast, NJA‐312low treatment upregulated IL17A in the skin, especially in keratinocytes and intradermal cells (Figure 6H).

Although no lung manifestations of aGvHD had been detected for either carrier‐ or NJA‐312low‐treated mice, strong IL17A staining was found in epithelial cells especially of NJA‐312low‐treated mice (Figure S1B). These data suggest that NJA‐312low treatment acted organ specifically, reducing tissue IL17A expression in liver and splenocytes but increasing it in skin and lung.

Our study demonstrated that siRNA targeting of CD40 coupled to β‐glucan enhanced survival and reduced organ damage in the liver and spleen, but not the skin by suppressing myeloid cell and IL17A‐expressing cell accumulation in lymphoid organs of mice with aGvHD. Compound treatment delayed T and B‐cell donor engraftment in lymphoid organs like the spleen and thymus, but T and B‐cell donor engraftment and chimerism in surviving mice (Figure 6I). NJA‐312 suppressed increases in CCR2+, CD11b+, and IL17A+ cells in the liver, spleen, and thymus, but not the skin.

## 4 DISCUSSION

Targeting the CD40‐CD40L dyad is a strategy to reduce aGvHD and induce long‐term engraftment after HSCT [9, 11, 22]. Here, we tested siCD40–SPG compounds that target CD40 in Dectin1+ cells mainly constituting monocytes, classical DCs, and others [19]. Other potential compound receptors are complement receptor 3 and TLR‐2/6 [31]. Recently, siCD40—SPG compounds have been shown to reduce CD40 expression on DC cells after injection in cynomolgus monkeys [19].

This is the first demonstration that siRNA can suppress the immune response after HSCT. The NJA‐312, NJA‐302, and to a lesser extent NJA‐515 compounds reduced aGvHD‐associated lethality and liver and lymphoid tissue destruction. NJA‐312 treatment delayed the initial donor T and B‐cell engraftment. Long‐term surviving mice showed stable chimerism of donor and recipient lymphocytes after NJA‐312 treatment. Dectin1 stimulation by β‐glucan enhances DC‐driven T helper type 17 cell (Th17) responses in vivo [32]. The cytokine profiles were not altered after compound injection into mice under steady‐state conditions or compound addition to cultured peripheral mononuclear cells (personal communication). But the complex inflammatory and immunological niche after allogeneic BMT changes the cytokine expression after compound addition as exemplified by IL17A expression.

IL17A producing CD4 T cells enhance macrophage differentiation and expedite the invasion of T cells (like CD4) and macrophages into GvHD organs [33]. High IL17A expression correlated with a higher influx of F4/80+ macrophages and CCR2+ cells, and more severe tissue damage in the spleen, liver, and thymus, which could be suppressed but not abolished by siCD40/SPG treatment. While NJA‐312 treatment reduced IL17A expression in the liver, spleen, and thymus, an increased number of CD3+, IL17A+, and CD11b+ inflammatory cells were found in the dermis of the skin.

Lower Dectin1 expression on macrophages and DCs [25] could be one reason for the reduced effectiveness of NJA‐312 to prevent skin aGvHD as less CD40 targeting siRNA could be delivered to skin DCs. The tissue expression pattern of Dectin1 might have contributed to the differences in the compound efficacy and the modulation of the local inflammatory response after compound administration. Furthermore, Langerhans cells, the major APC in the skin, are also sufficient for the induction of skin GvHD when all other APCs cannot prime donor T cells. The ineffectiveness of the compounds for skin GvHD could also be related to the β‐glucan receptors expressed on Langerhans cells. It is conceivable that Langerhans cells compared to DCs in other organ tissues, express fewer β‐glucan receptors, including CD40 or TLRs.

The number of compound injections pre‐BMT (NJA‐312low with two injections vs. NJA‐312high with three injections) generates a tolerance state in the host required for long‐term survival. Our data are consistent with earlier studies demonstrating that ex vivo blockade of CD4 T cells using anti‐CD40L antibody induces tolerance of CD4 T cells to host alloantigens and reduces GvHD while preserving T‐cell responses directed to those antigens not present during tolerization [34].

It is unclear that cytokines were responsible for the severe early bodyweight loss in allogenic BMT mice treated with high but not lower doses of NJA‐312. Initial induction of a Th17 response might have contributed to the bodyweight loss in NJA‐treated animals. IL17A, although not changing overall GvHD and graft versus tumor (GVT) activity, contributes to the early development of CD4‐mediated GvHD by promoting the production of proinflammatory cytokines [30] and might have contributed to the bodyweight loss in the early phase after aGvHD induction.

Essential for the clinical use of the siCD40/SPG compounds is that a Y238X polymorphism of the Dectin1 gene results in lower Dectin‐1 receptor expression on immune cells [35]. In addition, carriers of the Dectin1 Y238X polymorphism showed a more severe aGvHD and decreased Th17 responses [36].

Patients undergoing BMT receive drugs like cyclosporin A alone or combined with methotrexate to prevent T‐cell activation and aGvHD. The U.S. food and drug administration recently approved abatacept, a drug that targets another costimulatory pathway (CTLA4/CD80) to treat aGvHD. Further studies are required to optimize siCD40/SPG administration timing and test the compounds in combination with known drugs like cyclosporin A or methothrexate.

In conclusion, siCD40/SPG compounds improved immunological reconstitution and survival in murine aGvHD models and impaired tissue damage in extramedullar organs, but not the skin. Mechanistically siCD40–SPG compounds impaired CD11b+ and CCR2+ cell accumulation and reduced IL17A expression (Figure 6I). The use of PAMP receptors like Dectin1 for the entry of siRNA into inflammatory cells is intriguing as it seems to result in tissue‐specific modulation of the inflammatory niche. It seems to be a therapeutic strategy to block alloreactivity, but studies will be necessary to determine whether or not it compromises the graft versus tumor effects.

## AUTHOR CONTRIBUTIONS

*Data acquisition*: Beate Heissig, Yousef Salama, and Masatoshi Tateno. *Pathological examination and writing—review and editing*: Beate Heissig, Yousef Salama, Satoshi Takahashi, and Koichi Hattori. *Visualization*: Beate Heissig. *Supervision*: Beate Heissig. *Project administration*: Beate Heissig. *Funding acquisition*: Beate Heissig, Koichi Hattori, and Satoshi Takahashi. All authors have read and agreed to the published version of the manuscript.

## CONFLICT OF INTEREST

The authors declare they have no conflicts of interest.

## ETHICS STATEMENT

Animal procedures were approved by the institutional animal care committee of The Institute of Medical Science, the University of Tokyo, Japan.

## Supporting information

Supporting Information

Supporting Information

## Data Availability

Data sharing not applicable to this article as no datasets were generated or analyzed during the current study.
