# Supplementary figures and images for: siRNA against CD40 delivered via a fungal recognition receptor ameliorates murine acute graft‐versus‐host disease
Source: EJHaem. 2022 May 6;3(3):849–61. doi: 10.1002/jha2.439 (PMC9421973; doi:10.1002/jha2.439)

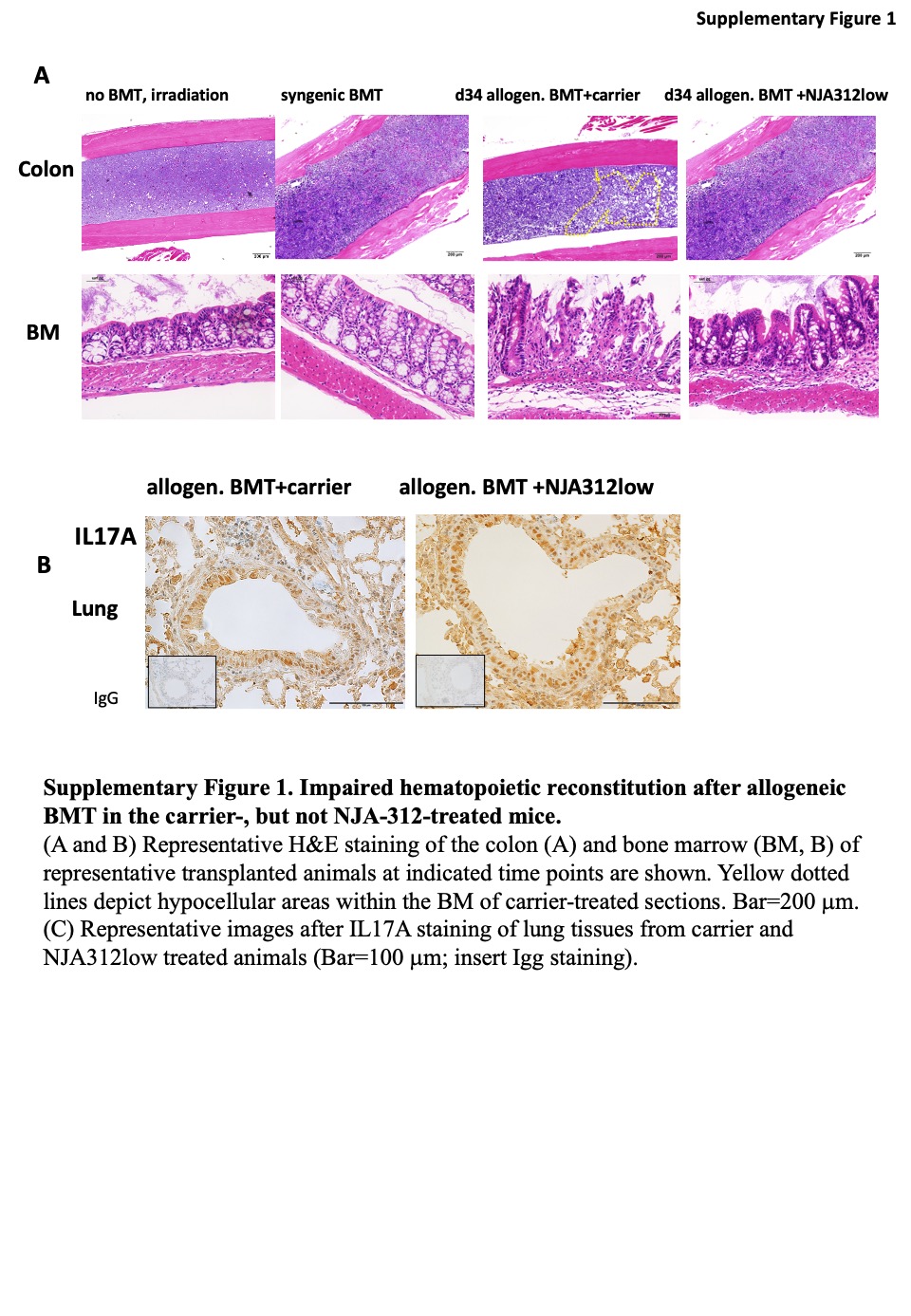

Supplement: Supplementary file 1 — Supporting Information [file JHA2-3-849-s001.jpeg]
