# Supplementary material for: siRNA against CD40 delivered via a fungal recognition receptor ameliorates murine acute graft‐versus‐host disease
Source: EJHaem. 2022 May 6;3(3):849–61. doi: 10.1002/jha2.439 (PMC9421973; doi:10.1002/jha2.439)
Supplement: Supplementary file 2 — Supporting Information [file JHA2-3-849-s002.docx]

**Supplementary Figure 1. Impaired hematopoietic reconstitution after allogeneic BMT in the carrier-, but not NJA-312-treated mice.**

(A and B) Representative H&E staining of the colon (A) and bone marrow (BM, B) of representative transplanted animals at indicated time points are shown. Yellow dotted lines depict hypocellular areas within the BM of carrier-treated sections. Bar=200 mm.

(C) Representative images after IL17A staining of lung tissues from carrier and NJA312low treated animals (Bar=100 mm; insert Igg staining).
